# Enhancement by Nano-Diamino-Tetrac of Antiproliferative Action of Gefitinib on Colorectal Cancer Cells: Mediation by EGFR Sialylation and PI3K Activation

**DOI:** 10.1007/s12672-018-0341-x

**Published:** 2018-09-05

**Authors:** Tung-Cheng Chang, Yu-Tang Chin, André Wendindondé Nana, Shwu-Huey Wang, Yu-Min Liao, Yi-Ru Chen, Ya-Jung Shih, Chun A. Changou, Yu-Chen SH Yang, Kuan Wang, Jacqueline Whang-Peng, Liang-Shun Wang, Steven C. Stain, Ai Shih, Hung-Yun Lin, Chih-Hsiung Wu, Paul J. Davis

**Affiliations:** 1 Graduate Institute of Clinical Medicine, College of Medicine, Taipei Medical University, Taipei, 11031 Taiwan; 2 Division of Colorectal Surgery, Department of Surgery, Shuang Ho Hospital, Taipei Medical University, New Taipei City, 23561 Taiwan; 3 Division of Colorectal Surgery, Department of Surgery, School of Medicine, College of Medicine, Taipei Medical University, Taipei, 11031 Taiwan; 4 Taipei Cancer Center, Taipei Medical University, Taipei, 11031 Taiwan; 5 The PhD program for Cancer Molecular Biology and Drug Discovery, College of Medical Science and Technology, Taipei Medical University, Taipei, Taiwan; 6 Core Facility Center, Office of Research and Development, Taipei Medical University, Taipei, 11031 Taiwan; 7 Department of Biochemistry and Molecular Cell Biology, College of Medicine, Taipei Medical University, Taipei, 11031 Taiwan; 8 Division of Hematology and Oncology, Department of Internal Medicine, Taipei Medical University Hospital, Taipei, 11031 Taiwan; 9 Integrated Laboratory, Center of Translational Medicine, Taipei Medical University, Taipei, 11031 Taiwan; 10 Joint Biobank, Office of Human Research, Taipei Medical University, Taipei, 11031 Taiwan; 11 Graduate Institute of Nanomedicine and Medical Engineering, College of Medical Engineering, Taipei Medical University, Taipei, 11031 Taiwan; 12 Taipei Cancer Center; Cancer Center, Wan Fang Hospital, Taipei Medical University, Taipei, Taiwan; 13 Department of Surgery, Shuang Ho Hospital, Taipei Medical University, No. 291, Zhongzheng Rd., Zhonghe, New Taipei City, 23561 Taiwan; 14 Department of Surgery, Albany Medical College, Albany, NY 12208 USA; 15 National Laboratory Animal Center, Taipei, 11599 Taiwan; 16 Pharmaceutical Research Institute, Albany College of Pharmacy and Health Sciences, Rensselaer, NY 12144 USA; 17 Traditional Herbal Medicine Research Center of Taipei Medical University Hospital, Taipei Medical University, Taipei, 11031 Taiwan; 18 TMU Research Center of Cancer Translational Medicine, Taipei Medical University, Taipei, 11031 Taiwan; 19 NanoPharmaceuticals LLC, Rensselaer, NY 12144 USA; 20 Department of Medicine, Albany Medical College, Albany, NY 12208 USA

## Abstract

Drug resistance complicates the clinical use of gefitinib. Tetraiodothyroacetic acid (tetrac) and nano-diamino-tetrac (NDAT) have been shown in vitro and in xenografts to have antiproliferative/angiogenic properties and to potentiate antiproliferative activity of other anticancer agents. In the current study, we investigated the effects of NDAT on the anticancer activities of gefitinib in human colorectal cancer cells. β-Galactoside α-2,6-sialyltransferase 1 (ST6Gal1) catalyzes EGFR sialylation that is associated with gefitinib resistance in colorectal cancers, and this was also investigated. Gefitinib inhibited cell proliferation of HT-29 cells (K-ras wild-type), and NDAT significantly enhanced the antiproliferative action of gefitinib. Gefitinib inhibited cell proliferation of HCT116 cells (K-ras mutant) only in high concentration, and this was further enhanced by NDAT. NDAT enhancedd gefitinib-induced antiproliferation in gefitinib-resistant colorectal cancer cells by inhibiting ST6Gal1 activity and PI3K activation. Furthermore, NDAT enhanced gefitinib-induced anticancer activity additively in colorectal cancer HCT116 cell xenograft-bearing nude mice. Results suggest that NDAT may have an application with gefitinib as combination colorectal cancer therapy.

## Introduction

New therapeutic approaches are needed for metastatic colon cancer. Certain molecular targets have attracted attention in this form of cancer. Epidermal growth factor (EGF) plays an important role in embryonic growth and development. The EGF receptors (EGFRs) are a family of receptors that include HER1 (erb-B1), HER2 (erb-B2), and HER3 (erb-B3) [1]. Normal EGFR activity is required for the establishment of intestinal tumors in the APC-mediated initiation of intestinal tumorigenesis [2]. Overexpression of EGFR is involved in the development of several types of cancers including colorectal cancer [3, 4]. Low tumor EGFR expression in patients with colorectal cancer is associated with low tumor metastasis risk and better survival [5].

There is also a crosstalk between EGFR signaling and the Wnt-β-catenin pathway. While the former activates β-catenin via the receptor tyrosine kinase-PI3K/Akt pathway, the latter can activate EGFR signaling via transmembrane Frizzled receptor [6, 7]. EGFR is able to form a complex with β-catenin, increasing the invasiveness and frequency of metastasis of cancer cells [6]. Mutations of APC, K-ras, and β-catenin genes have been shown to be early events in tumorigenesis colon cancer [8, 9], but whether relationships exist among these events is unclear.

β-Galactoside α2,6-sialyltransferase (ST6Gal1) catalyzes α2,6 sialylation of N-glycan. Functional ST6Gal1 on EGFR has been shown to be highly correlated with colon cancer progression and metastasis [10]. Increased α2,6 sialylation may also enhance radioresistance in colon cancer [10]. The anticancer activity of a chemotherapeutic tyrosine kinase inhibitor, gefitinib (Iressa®), is augmented in ST6Gal1-deficient colon cancer cells. In contrast, overexpression of ST6Gal1 has been found to decrease the cytotoxic effect of gefitinib. Such results suggest that sialylation of EGFR affects EGF-mediated cell growth and induces chemoresistance to gefitinib in colon cancer cells.

Gefitinib is a selective inhibitor of EGFR tyrosine kinase [11] and has been used in the treatment of colorectal cancer and other types of cancers, either as monotherapy or in combination with other agents [12]. Gefitinib resistance in cancers depends on the activation of specific signal transduction pathways, e.g., ERKs and PI3K [13]. Gefitinib disrupts K-ras/PI3K and K-ras/Raf complexes in human nonsmall cell lung cancer (NSCLC) Calu3 cells, but not in Calu3 K-ras mutant cells [12, 14]. Cell K-ras mutation is associated with resistance to gefitinib therapy [15]. The consequences of gefitinib-inhibited EGFR activity are dephosphorylation of EGFR, HER2, and HER3; the dissociation between HER3 and PI3K; and decreased Akt activity [16]. EGFR mutation can also affect the sensitivity of colorectal cancers to gefitinib, but the effect is not consistent [17].

Gefitinib has been shown to inhibit human chondrosarcoma proliferation and metastasis by induction of cell cycle arrest and a decrease of migration capacity. Gefitinib also reduces the expression of metastasis-related proteins, such as basic fibroblast growth factor (bFGF) and matrix metalloproteinases-2 (MMP-2) and MMP-9 [18]. Gefitinib has been combined with other cancer chemotherapeutic agents in the management of various cancers [19–22]. What is clear is that gefitinib affects a number of the cancer cell therapeutic targets mentioned above, yet resistance to this tyrosine kinase inhibitor (TKI) develops. In the current report, we describe a new treatment strategy that restores responsiveness to gefitinib.

The deaminated analogue of L-thyroxine, tetraiodothyroacetic acid (tetrac), and its nanoparticulate derivative, nano-diamino-tetrac (NDAT), have been shown to inhibit cancer cell proliferation and tumor-relevant angiogenesis by differential modulation of the expression of a substantial number of genes involved in apoptosis and antiangiogenesis [23–25]. Tetrac and NDAT are not cytotoxic when incubated with nonmalignant cells [24, 26, 27]. We describe here the efficacy of the combination of NDAT and gefitinib in human colorectal cancer cell lines and identify proliferative, pro-apoptotic genes and metastasis-linked genes whose expression is affected by this chemotherapeutic combination. We found that NDAT blocked ST6Gal1-induced sialylation of EGFR and consequent PI3K activation, which are essential for proliferation of cancer cells in both K-ras wild-type (wt) and K-ras mutant colorectal cancer cells. Xenograft studies also confirmed that NDAT enhanced gefitinib-induced anticancer activity additively in HCT116 colorectal cancer xenograft-bearing mice. Combination NDAT-gefitinib treatment has anticancer potential that surpasses the effect of each agent taken individually.

## Materials and Methods

### Cell Lines

Human colorectal cancer cell lines, HT-29 (ATCC® HTB-38™) and HCT116 (ATCC® CCL-247™) cells, were purchased from American Type Culture Collection (ATCC, Manassas, VA, USA) by the Bioresource Collection and Research Center (BCRC, Hsinchu, Taiwan). These two cell lines have been tested and authenticated (including isoenzyme analysis, mycoplasma test, cytogenetics test, tumorigenic test, and receptor expression test) by BCRC. These cell lines were then purchased from BCRC by H.Y Lin’s lab and and passaged for less than 6 months after thawing and maintaining them for further study in RPMI 1640 Medium (Life Technologies Corp., Carlsbad, CA, USA) supplemented with 10% fetal bovine serum (FBS) and under incubation conditions of 5% CO_2_ at 37 °C. Before these treatments, cells were placed in serum-free medium for 24-h starvation.

### Cell Viability Assay

HCT116 cells and HT-29 cells were plated at a density of 10^4^ cells/well in 96-well plates. Cell viability was determined with the CyQUANT® NF Cell Proliferation Assay Kit (Molecular Probes, Eugene, OR, USA) at 96 h after treatment. Briefly, medium was removed, and cells were incubated with CyQUANT® NF reagent for 1 h at 37 °C according to the manufacturer’s instructions. Plates were then analyzed using a microplate reader (Varioskan™ Flash Multimode Reader, Thermo Scientific, Waltham, MA, USA) (excitation, 485 nm; emission, 530 nm). Data are expressed as the mean percentage of cell viability ± SD.

### Quantitative RT-PCR (qPCR)

Total RNA was extracted, and genomic DNA was eliminated with illustra RNAspin Mini RNA Isolation Kit (GE Healthcare Life Sciences, Buckinghamshire, UK). One microgram of DNase I-treated total RNA was reverse-transcribed with RevertAid H Minus First Strand cDNA Synthesis Kit (Life Technologies Corp.) into cDNA and used as the template for real-time PCR reactions and analysis. The real-time PCR reactions were performed using QuantiNova™ SYBR® Green PCR Kit (QIAGEN, Valencia, CA, USA) on CFX Connect™ Real-Time PCR Detection System (Bio-Rad Laboratories, Inc., Hercules, CA, USA). This involved an initial denaturation at 95 °C for 5 min, followed by 40 cycles of denaturing at 95 °C for 5 s and combined annealing/extension at 60 °C for 10 s, as described in the manufacturer’s instructions. The primer sequences were shown as follows: *Homo sapiens* proliferating cell nuclear antigen (*PCNA*), forward 5′-TCTGAGGGCTTCGACACCTA-3′ and reverse 5′-TCATTGCCGGCGCATTTTAG-3′ (Accession No. BC062439.1); *Homo sapiens* cyclin D1 (*CCND1*), forward 5′-CAAGGCCTGAACCTGAGGAG-3′ and reverse 5′-GATCACTCTGGAGAGGAAGCG-3′ (Accession No. NM_053056); *Homo sapiens* v-myc avian myelocytomatosis viral oncogene homolog (*c-MYC*), forward 5′-TTCGGGTAGTGGAAAACCAG-3′ and reverse 5′-CAGCAGCTCGAATTTCTTCC-3′ (Accession No. NM_002467); *Homo sapiens* tumor protein p53 (*p53*), forward 5′-AAGTCTAGAGCCACCGTCCA-3′ and reverse 5′-CAGTCTGGCTGCCAATCCA-3′ (Accession No. NM_000546.5); *Homo sapiens* caspase 2, apoptosis-related cysteine peptidase (*CASP2*), forward 5′-GCATGTACTCCCACCGTTGA-3′ and reverse 5′-GACAGGCGGAGCTTCTTGTA-3′ (Accession No. NM_032982.3); *Homo sapiens* BCL2-associated agonist of cell death (*BAD*), forward 5′-CTTTAAGAAGGGACTTCCTCGCC-3′ and reverse 5′-AAGTTCCGATCCCACCAGGA-3′ (Accession No. NM_032989.2); *Homo sapiens* matrix metallopeptidase 2 (*MMP-2*), forward 5′-ATCCAGACTTCCTCAGGCGG-3′ and reverse 5′-CCTGGCAATCCCTTTGTATGTT-3′ (Accession No. NM_004530.5); *Homo sapiens* matrix metallopeptidase 9 (*MMP-9*), forward 5′-TGTACCGCTATGGTTACACTCG-3′ and reverse 5′-GGCAGGGACAGTTGCTTCT-3′ (Accession No. NM_004994.2); *Homo sapiens* vascular endothelial growth factor A (*VEGF-A*), forward 5′-TACCTCCACCATGCCAAGTG-3′ and reverse 5′-GATGATTCTGCCCTCCTCCTT-3′ (Accession No. NM_001171623.1); *Homo sapiens* ST6 beta-galactosamide alpha-2,6-sialyltranferase 1 (*ST6GAl1*), forward 5′-TGCAGTCATCTGTGTGTGGAA-3′ and reverse 5′-CACCTGGAATTCCTTGGTTTGC-3′ (Accession No. BC031476.1); *Homo sapiens* 18S rRNA gene (*18S*), forward 5′-GTAACCCGTTGAACCCCATT-3′ and reverse 5′-CCATCCAATCGGTAGTAGCG-3′ (Accession No. M10098). Calculations of relative gene expression (normalized to 18S reference gene) were performed according to the ΔΔCT method. Fidelity of the PCR reaction was determined with melting temperature analysis.

### Western Blotting

To examine the signaling pathways involved in the antiproliferative effects of NDAT, gefitinib, and their combination, we performed Western blot analyses to quantify the protein expression levels of pPI3K(p85) and ST6GAl1 in the total cell lysates of HCT116 cells that were treated with NDAT, gefitinib, or their combination. Protein samples were resolved on a 10% sodium dodecyl sulfate polyacrylamide gel (SDS-PAGE). A 20 μg quantity of protein was loaded in each well with 5× sample buffer, and the protein samples were resolved with electrophoresis at 100 V for 2 h. The resolved proteins were transferred from the polyacrylamide gel to Millipore Immobilon-PSQ Transfer PVDF membranes (Millipore, Billerica, MA, USA) with the Mini Trans-Blot® Cell (Bio-Rad Laboratories). The membranes were blocked with a solution of 2% FBS in Tris-buffered saline. The membranes were incubated with primary antibodies to pPI3K(p85) (Cell Signaling Technology, Inc., Beverly, MA, USA), ST6Gal1 (Millipore), and GAPDH (GeneTex International Corp., Hsinchu City, Taiwan) at 4 °C overnight and washed, and the proteins were detected with HRP-conjugated secondary antibodies and Immobilon™ Western HRP Substrate Luminol Reagent (Millipore). Images of the Western blots were visualized and recorded with an Amersham Imager 600 (GE Healthcare, Chicago, IL, USA). The blots were quantified using ImageJ software (National Institutes of Health, Bethesda, MD, USA).

### Confocal Microscopy

HCT116 cells exponentially growing on sterilized cover glass slides (Paul Marienfeld GmbH & Co. KG, Lauda-Königshofen, Germany) were treated with gefitinib, with or without NDAT or PI3K inhibitor (LY294002) (Selleck Chemicals). The cells were immediately fixed with 4% paraformaldehyde in phosphate-buffered saline (PBS) for 20 min. Fluorescein-labeled *Sambucus nigra* lectin (FITC-SNA, Vector Laboratories, Burlingame, CA, USA) that preferentially binds to α2,6-linked sialic acid structure was used to investigate the product of ST6Gal1 after these treatments. Cells on the slides were incubated with anti-EGFR antibody (GeneTex) overnight at 4 °C and then incubated with Alexa Fluor®-647-conjugated secondary antibody (Abcam, Cambridge, UK) for 1 h at room temperature (kept in the dark). Cells were then incubated with FITC-SNA (Vector Laboratories) for 15 min at room temperature (kept in the dark). All slides were mounted in EverBrite Hardset mounting medium with DAPI (Biotium, Hayward, CA, USA). The fluorescent signals from EGFR and FITC-SNA were recorded and analyzed with TCS SP5 Confocal Spectral Microscope Imaging System (Leica Microsystems, Wetzlar, Germany). The figures shown are representative of four fields for each experimental condition. Nuclei were defined with DAPI staining.

### Xenografts

Nude mice (BALB/cAnN.Cg-Foxn1nu/CrlNarl, male) were purchased from National Laboratory Animal Center (Taipei, Taiwan), housed in a reserved, pathogen-free facility, and handled in accordance with the protocols approved by the Institutional Animal Care and Use Committee of the National Defense Medical Center, Taipei, Taiwan (IACUC-15-340). Mice were acclimated to the vivarium for 1 week prior to their use according to study protocols. Up to four animals were housed in a cage under conventional conditions and fed chow and water ad libitum. For xenograft implantation, mice were anesthetized with a mixture of ketamine and xylazine (120 mg/kg ketamine, 10 mg/kg xylazine). Each mouse was inoculated subcutaneously with aliquots of 1 × 10^6^ HCT116 cells/100 μl Matrigel (BD Matrigel™ Basement Membrane Matrix) (BD Biosciences, San Jose, CA, USA) on each flank, using a 26-gauge needle on a tuberculin syringe. After inoculation, the animals were treated intraperitoneally with solvent (PBS), gefitinib (in PBS containing 0.5% Tween 80, 10 mg/kg/twice a week), NDAT (in PBS, 1 mg/kg/twice a week), or the combination for 5 weeks. Tumor volumes were measured twice a week using digital calipers, and the volume calculated as (length × width × width)/2 and expressed as cubic millimeters (mm^3^). The -fold tumor volume change was calculated from final measured volume divided by the the initial measured volume. These results were averaged and variability expressed as mean ± SE. After treatment for 5 weeks, all animals were sacrificed and the tumors were resected.

### Statistical Analysis

Western blotting densities, gene expression of qPCR, and tumor volume changed fold were analyzed with IBM SPSS Statistics software version 19.0 (SPSS Inc., Chicago, IL, USA). Student’s *t* test was conducted and considered significant at *p* < 0.05 (*, #, &, $), 0.01 (**, ##, &&, $$), and 0.001 (***, ###, &&&, $$$).

## Results

### Gefitinib induces moderate antiproliferation that is enhanced by NDAT in colorectal cancer cell cultures

**Fig. 1:**
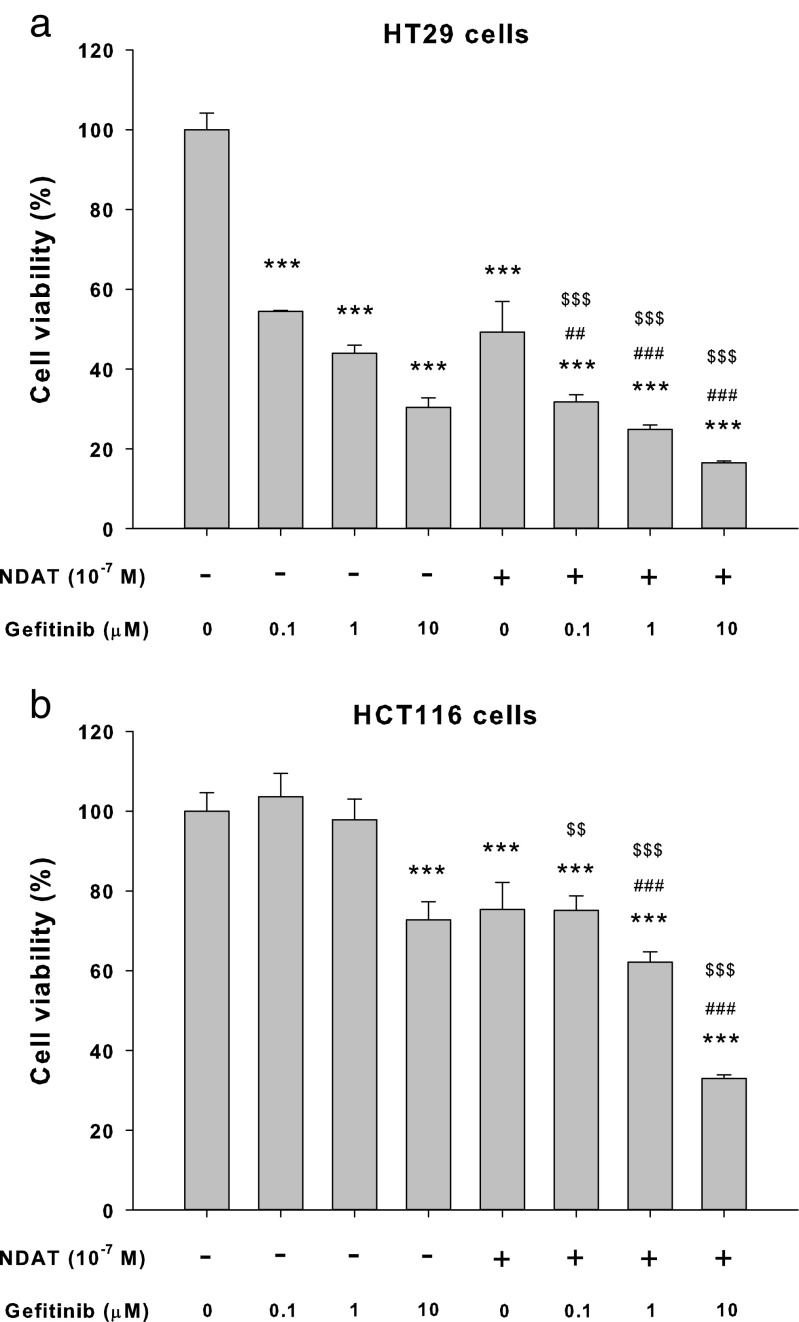
NDAT enhances gefitinib-induced antiproliferation in human colorectal cancer cell lines. **a** HT-29 cells and **b** HCT116 cells were seeded in 96-well plates and treated with different concentrations of gefitinib, with 10^−7^ M of NDAT, or their combination. Medium with drugs was refreshed daily for 144 h. Cell proliferation was examined with CyQUANT® NF Cell Proliferation Assay Kit. Number of independent experiments (*N*) = 6. Data are expressed as mean ± SD; compared to the untreated control, *p < 0.05, **p < 0.01, ***p < 0.001, compared with control; ^#^*p* < 0.05, ^##^*p* < 0.01, ^###^*p* < 0.001, compared with NDAT; ^$^*p* < 0.05, ^$$^*p* < 0.01, ^$$$^*p* < 0.001, compared with Gefitinib

Colorectal cancer HCT116 and HT-29 cells were treated with different concentrations of gefitinib for 96 h. Gefitinib inhibited cell proliferation in a concentration-dependent manner. However, the concentration used was higher than that of other cancer cell studies [28]. Tetrac, an L-thyroxine metabolite, has been shown to induce antiproliferation in various cancer cell lines [29]. To define possible enhancement of efficacy of gefitinib-induced antiproliferation in colorectal cancer cells, a nanoparticulate derivative of tetrac, NDAT, was used in combination with gefitinib. Colorectal cancer HT-29 cells were treated with 10^−7^ M of NDAT in the presence or absence of different concentrations of gefitinib. Results showed that NDAT enhanced the antiproliferation induced by gefitinib and that the combination significantly increased antiproliferation compared to each agent, alone (Fig. 1a). Similar results were observed in another colorectal cancer cell line, HCT116 (Fig. 1b), which contains a mutant K-ras and is resistant to chemotherapy.

### NDAT Enhances Gefitinib-Induced Downregulation of Proliferative Genes in Colorectal Cancer Cell Lines

**Fig. 2:**
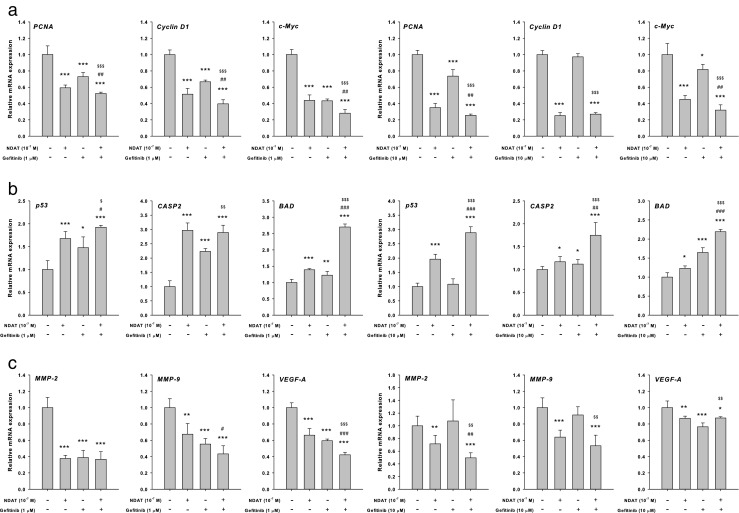
NDAT enhances gefitinib-regulated gene expression in colorectal cancer cell lines. Colorectal HT-29 cells (left-hand panel) and HCT116 cells (right-hand panel) were treated with gefitinib, NDAT, or gefitinib in combination with NDAT for 24 h. Cells were harvested and total RNA was extracted. qPCR was conducted for **a** proliferative genes, **b** pro-apoptotic genes, and **c** metastatic genes as described in the “Materials and Methods.” *N* = 6. Data are expressed mean ± SD; compared to the untreated control, *p < 0.05, **p < 0.01, ***p < 0.001, compared with control; ^#^*p* < 0.05, ^##^*p* < 0.01, ^###^*p* < 0.001, compared with NDAT; ^$^*p* < 0.05, ^$$^*p* < 0.01, ^$$$^*p* < 0.001, compared with Gefitinib

To evaluate the functions of NDAT, gefitinib, and their combination in wt K-ras HT-29 cells, mRNA expression profiles of the proliferative genes *PCNA*, *cyclin D1*, and *c-Myc* were studied (Fig. 2a). The combination of NDAT (10^−7^ M) and gefitinib (1 μM) significantly reduced mRNA expression of proliferative genes *PCNA*, *cyclin D1*, and *c-Myc* while NDAT or gefitinib, each alone, also decreased expression of these genes. Parallel studies were conducted in K-ras mutant HCT116 cells. There was a modest effect on the expression of *PCNA* and *c-Myc* in 10 μM gefitinib-treated cells. The expression of *cyclin D1* was not affected by gefitinib treatment. However, expression of these genes was inhibited by 10^−7^ M NDAT treatment. Furthermore, the synergistic effects were significant for the expression of *PCNA* and *c-Myc*, compared to cells treated with gefitinib only.

### NDAT Enhances Gefitinib-Induced Expression of Pro-Apoptotic Genes in Colorectal Cancer Cell Lines

The effects of NDAT, gefitinib, and their combination on the expression profiles of the pro-apoptotic genes *p53*, *CASP2*, and *BAD* were evaluated in wt K-ras HT-29 cells (Fig. 2b). NDAT (10^−7^ M) or gefitinib (1 μM), alone, induced the expression of *p53*, *CASP2*, and *BAD*. The combination of NDAT (10^−7^ M) and gefitinib (1 μM) significantly increased the expression of *p53*, *CASP2*, and *BAD*. Parallel studies were conducted in K-ras mutant HCT 116 cells. NDAT induced the expression of *p53*, *CASP2*, and *BAD* significantly. There was a smaller effect on the expression of p53 in 10 μM gefitinib-treated cells, but the gefitinib-induced expression of *CASP2* and *BAD* was significant. Combination drug treatment resulted in enhanced expression of all three pro-apoptotic genes examined.

### NDAT Enhances Gefitinib-Induced Downregulation of the Metastatic Genes in Colorectal Cancer Cell Lines

In colorectal cancer HT-29 cells, treatment with gefitinib reduced the expression of *MMP-2*, *MMP-9*, and *VEGF-A,* which are involved in cancer cell invasiveness (Fig. 2c). Both NDAT and gefitinib significantly inhibited expression of *MMP-2*, *MMP-9* and *VEGF-A*. Expression of these genes, except for *VEGF-A*, was not further inhibited by combination treatment. On the other hand, NDAT blocked the expression of *MMP-2*, *MMP-9*, and *VEGF-A* in mutant HCT116 cells, whereas gefitinib inhibited the expression of *VEGF-A*, but not that of *MMP-2* and *MMP-9*. The combination treatment reduced the expression of *MMP-2* and *MMP-9* significantly.

### NDAT Inhibits the ST6Gal1-Catalyzed Sialylation of EGFR and PI3K Activation in Colorectal Cancer Cells

**Fig. 3:**
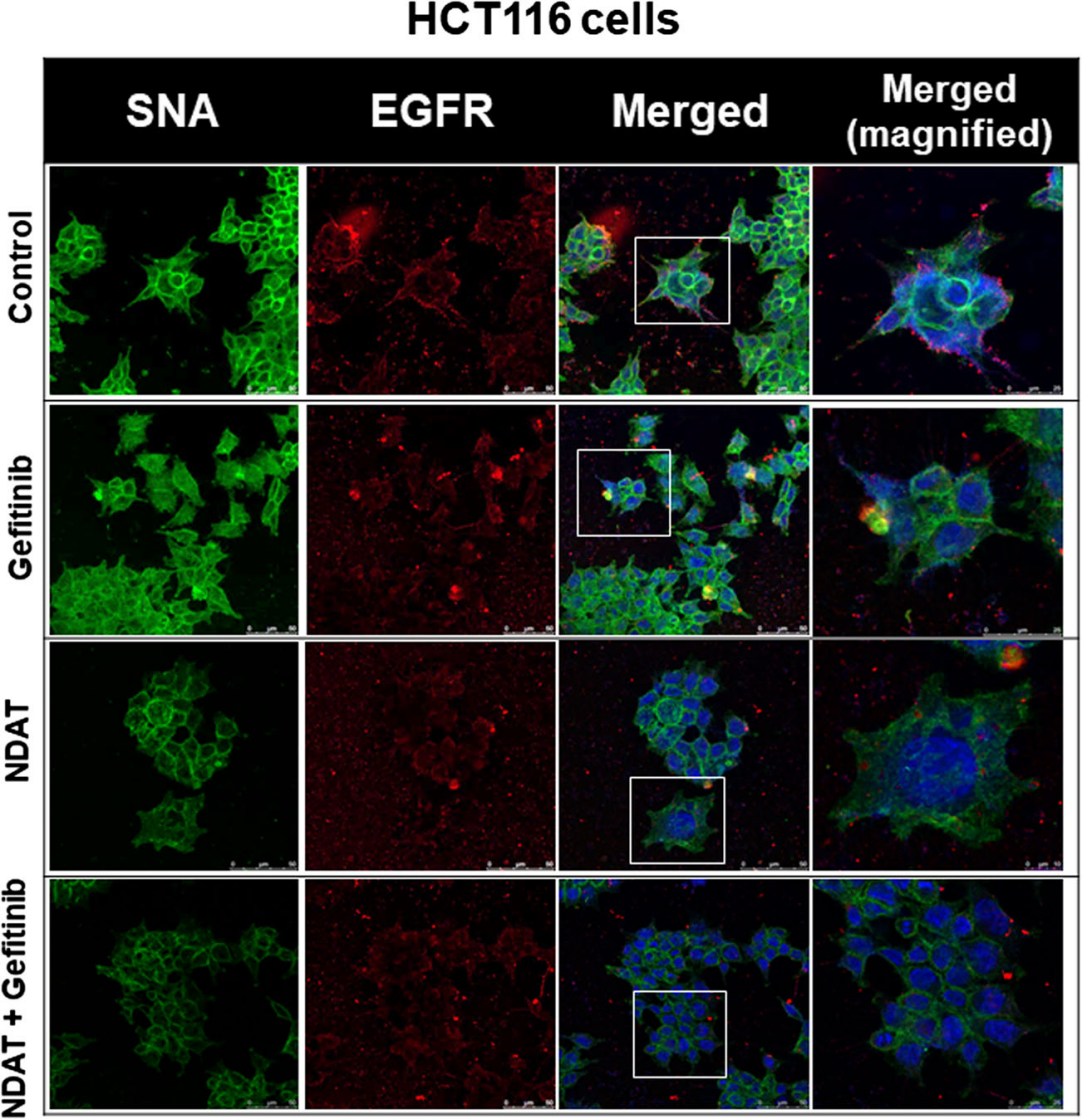
NDAT inhibits the ST6Gal1-catalyzed sialylation in colorectal cancer cells. HCT116 cells were seeded and grown on sterilized cover glass slides and then were treated with gefitinib (1 μM), with or without NDAT (10^−7^ M) for 24 h. The cells were immediately fixed and FITC-SNA was applied to bind to α2,6-linked sialic acid structure. Confocal microscopy was conducted to examine ST6Gal1-catalyzed α2,6 sialylation (green color) and EGFR expression (red color) in colorectal cancer cells

Gefitinib inhibits sialylation to induce antiproliferation. Therefore, we examined if NDAT and gefitinib were able to inhibit sialylation of EGFR in HCT116 cells. Results indicated that gefitinib did not affect the sialylation of EGFR; however, NDAT and the combination reduced more sialylation of EGFR in HCT116 cells (Fig. 3). NDAT (10^−7^ M) inhibited α2,6 sialylation. Gefitinib at 1 μM did not reduce ST6Gal1-induced α2,6 sialylation. NDAT enhanced additively the effect in the combination with different concentrations of gefitinib.

**Fig. 4:**
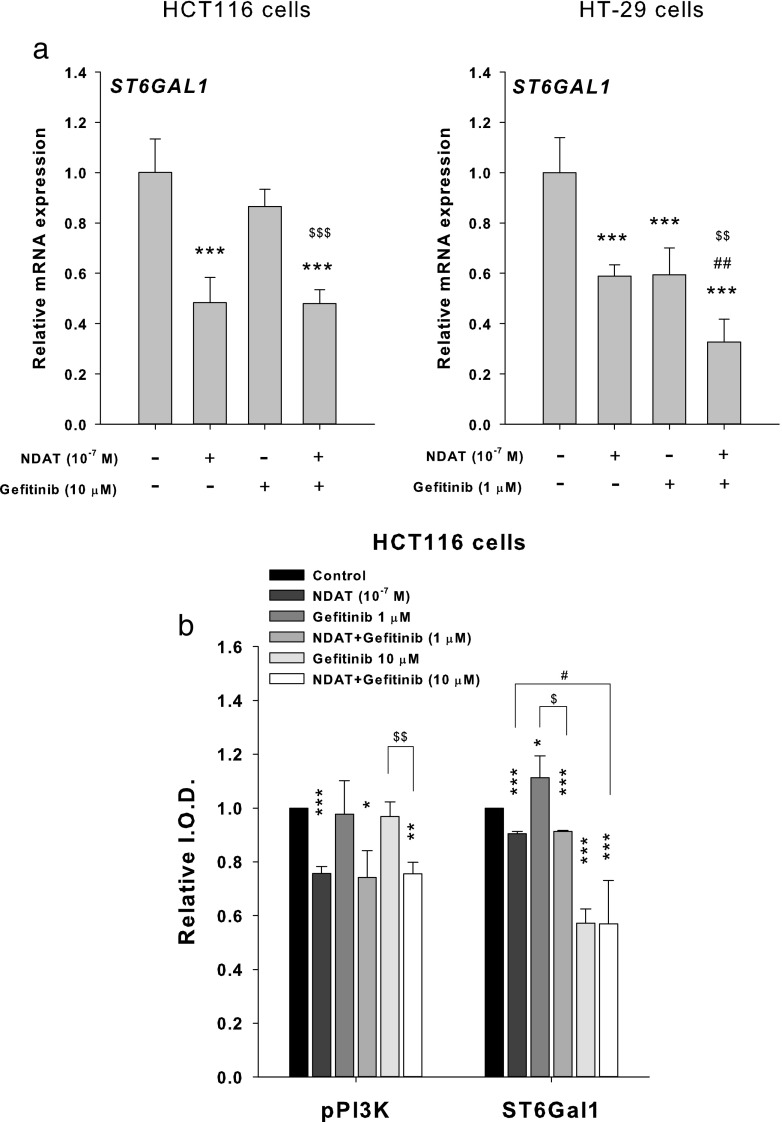
NDAT inhibits ST6Gal1 expression and PI3K activity in colorectal cancer cells. **a** HT-29 cells (left-hand panel) and HCT116 cells (right-hand panel) were treated with gefitinib in the presence or absence of NDAT for 24 h. Total RNA was harvested, and qPCR was conducted for ST6Gal1 gene expression. *N* = 3. **b** HCT116 cells were treated with gefitinib, NDAT, or the combination for 24 h. Cells were harvested, and immunoblotting analyses were conducted with antiphosphorylated PI3K or anti-ST6Gal1 antibody. GAPDH was used as internal loading control. *N* = 4. Data are expressed mean ± SD; compared to the untreated control, *p < 0.05, **p < 0.01, ***p < 0.001, compared with control; ^#^*p* < 0.05, ^##^*p* < 0.01, ^###^*p* < 0.001, compared with NDAT; ^$^*p* < 0.05, ^$$^*p* < 0.01, ^$$$^*p* < 0.001, compared with Gefitinib

We further investigated if NDAT affected the expression of ST6Gal1. Studies were then conducted to determine whether NDAT and gefitinib affected the expression of ST6Gal1. HT-29 cells and HCT116 cells were treated with gefitinib in the presence or absence of NDAT for 24 h. Gefitinib inhibited the mRNA expression of ST6Gal1 in HT-29 cells, but not in HCT116 cells (Fig. 4a). On the other hand, NDAT inhibited ST6Gal1 expression in both colorectal cancer cell cultures (Fig. 4a). We further examined the effects of NDAT and gefitinib on the accumulation of α2,6-sialylated protein. NDAT reduced the α2,6-sialylated protein in HCT116 cells. Gefitinib at 1 μM enhanced, but at 10 μM reduced, the accumulation of α2,6-sialylated protein in the HCT116 cells (Fig. 4b). NDAT reduced the accumulation of α2,6-sialylated protein when it was co-incubated with 1 μM gefitinib but not with 10 μM gefitinib. These results showed that gefitinib might affect the expression and accumulation of α2,6-sialylated protein that causes cell drug resistance.

Persistent activity of the PI3K/Akt and/or Ras/ERK pathways is associated with gefitinib resistance of NSCLC cell lines [30]. The anticancer effect of the EGFR kinase inhibitor, gefitinib, has been shown to be increased in ST6Gal1-deficient colon cancer cells [10]. In the current studies, NDAT, but not gefitinib, inhibited PI3K activity in K-ras mutant HCT116 cells (Fig. 4b).

**Fig. 5:**
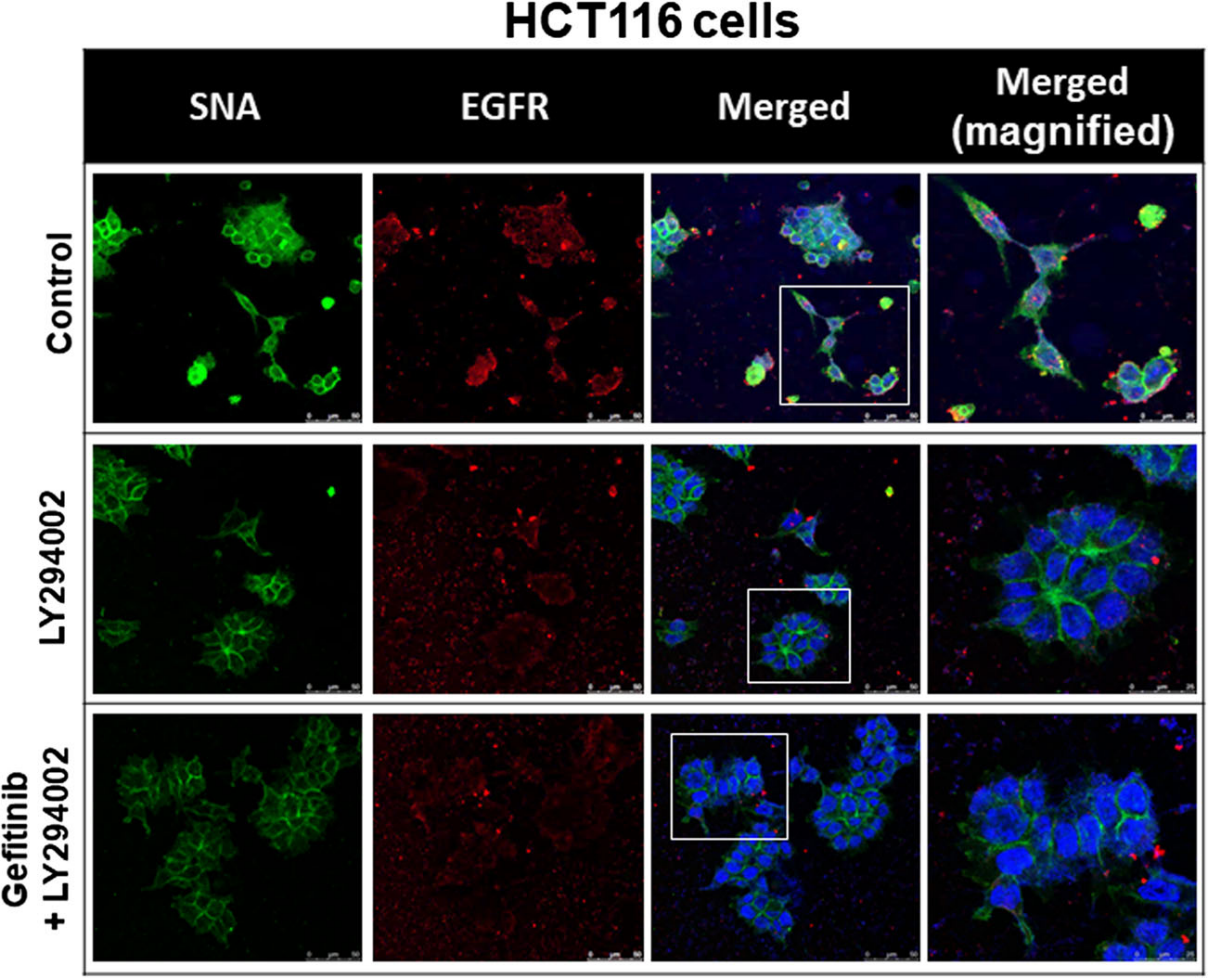
NDAT inhibits PI3K activity in colorectal cancer cells. Colorectal cancer HCT116 cells were seeded and grown on sterilized cover glass slides and then were treated with a PI3K inhibitor (LY294002, 10 μM) with or without gefitinib (1 μM) for 24 h. The cells were immediately fixed and FITC-SNA was applied to bind to α2,6-linked sialic acid structure. Confocal microscopy was used to examine ST6Gal1-catalyzed α2,6 sialylation (green color) and EGFR expression (red color) in colorectal cancer cells

**Fig. 6:**
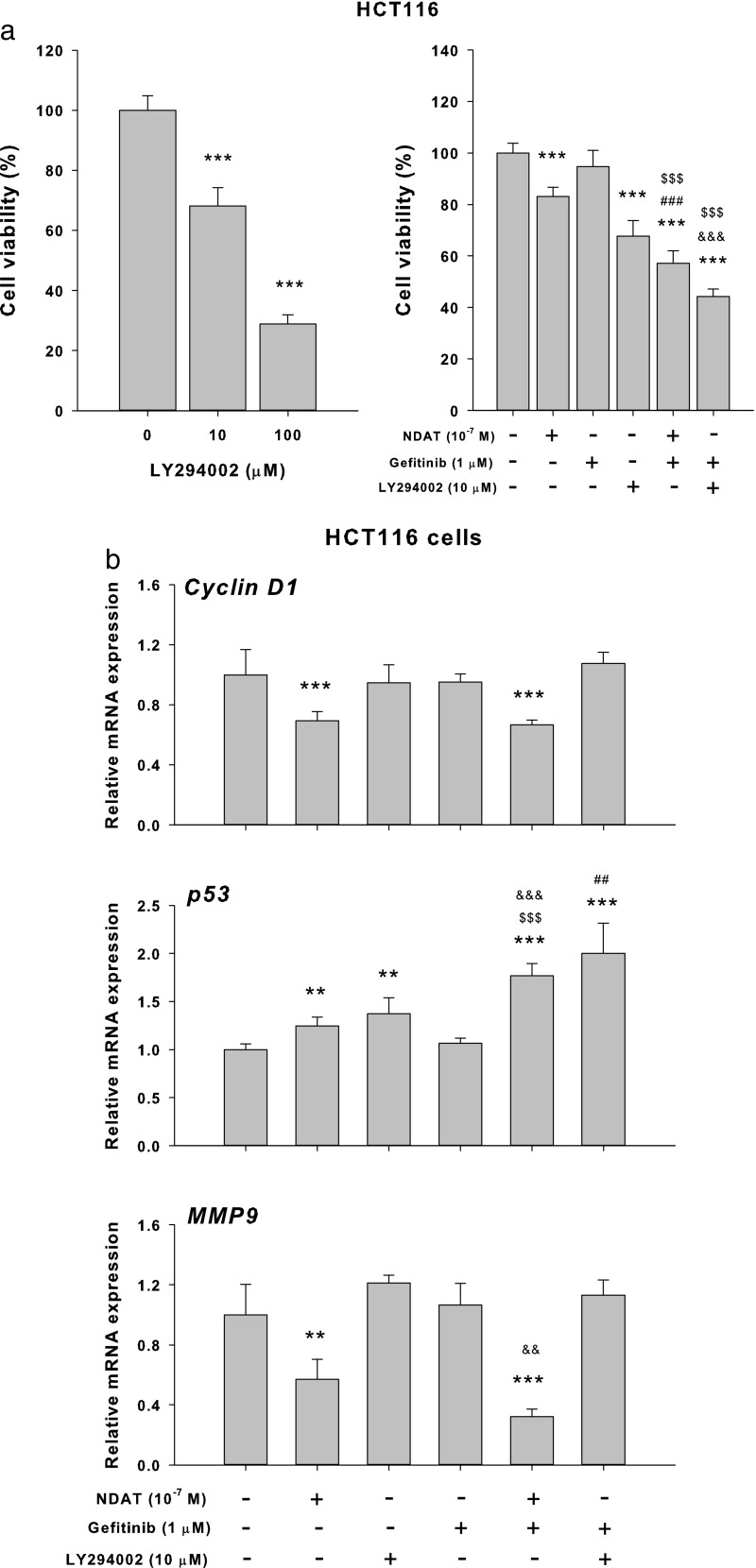
Inhibition of PI3K activity enhances the potentiating effect of NDAT on gefitinib-induced antiproliferation in colorectal cancer cells. **a** HCT116 cells were treated with different concentration of LY294002 for 144 h with refreshed medium containing LY294002 daily. Cells were treated with NDAT, gefitinib, and their combination with or without LY294002 (10 μM) for 144 h. Cell viability was evaluated with CyQUANT® NF Cell Proliferation Assay Kit (*N* = 6). **b** HCT116 cells were treated with NDAT, gefitinib, and their combination with or without LY294002 (10 μM) for 24 h (*N* = 4). Total RNA was extracted, and qPCR was conducted for cyclin D1, p53, and MMP-9. Data are expressed as mean ± SD; compared to the untreated control, *p < 0.05, **p < 0.01, ***p < 0.001, compared with control; ^#^*p* < 0.05, ^##^*p* < 0.01, ^###^*p* < 0.001, compared with NDAT; ^$^*p* < 0.05, ^$$^*p* < 0.01, ^$$$^*p* < 0.001, compared with Gefitinib

Results shown in Fig. 4 indicated that NDAT inhibited the constitutively activated PI3K in colorectal cancer cells. Therefore, we examined if the PI3K inhibitor, LY294002, was able to substitute the inhibitory effect of NDAT to reduce the sialylation of EGFR in HCT116. Results indicated that LY294002 affected the sialylation of EGFR and that the combination further reduced the sialylation of EGFR in HCT116 cells (Fig. 5). In addition, the potentiation by NDAT of the inhibitory effect of gefitinib on the cell viability (Fig. 6a) and the expression of proliferative genes was reproduced with the PI3K inhibitor (LY294002) and its combination with gefitinib in HCT116 cells (Fig. 6b).

### NDAT Enhances Gefitinib Antitumor Activity in HCT116 Xenograft Mouse Model

**Fig. 7:**
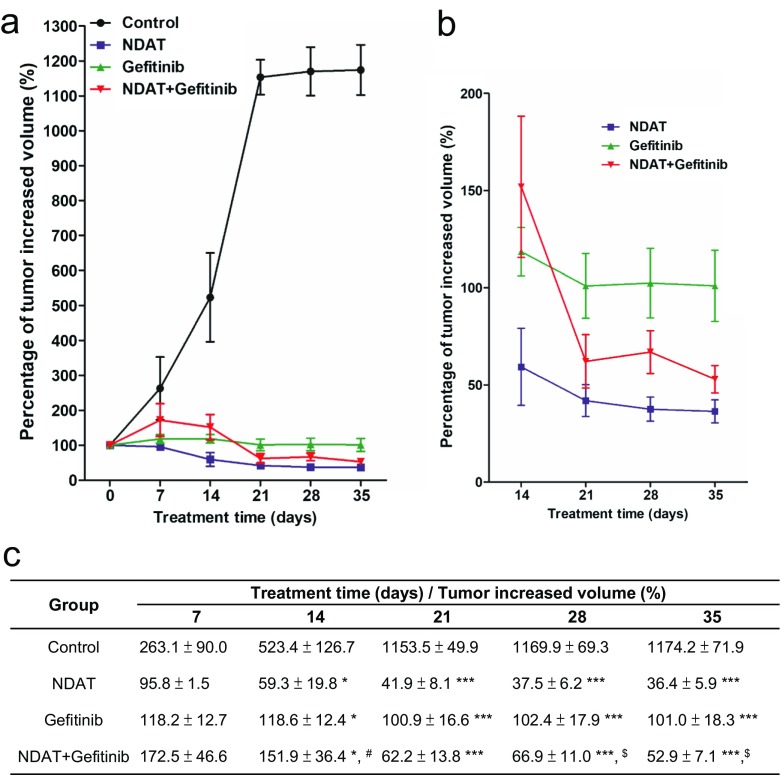
NDAT and gefitinib inhibit tumor growth in HCT116 xenografts. After HCT116 cell inoculation, the mice were treated intraperitoneally with solvent (PBS), gefitinib, NDAT, or their combination for 5 weeks. Tumor volumes were measured twice a week. The size of tumor was measured by using digital calipers, and the volume calculated as (length × width × width)/2 and expressed as cubic millimeters (mm^3^). **a** The percentage of tumor increased volume was calculated from 0, 7, 14, 21, 28, and 35 days’ measured volume divided by the first measured volume (0 day). **b** Data from **a** is shown as an expanded graph for days 14, 21, 28 and 35. **c** Results were averaged and variability expressed as mean ± SE, detailed statistical results are listed in the table. Compared to the untreated control, *p < 0.05, **p < 0.01, ***p < 0.001; compared to NDAT alone, ^#^*p* < 0.05; compared to gefitinib alone, ^$^*p* < 0.05

Studies of HCT116 cells xenografts in the nude mouse model also indicated that NDAT, alone and in combination with gefitinib, reduced PI3K activity, total amount of ST6Gal1 protein, and tumor size compared to single agent gefitinib-treated tumor (Fig. 7). NDAT enhanced gefitinib-induced antiproliferation in vivo. To investigate the potentiating effect of NDAT on the anticancer effect of gefitinib in vivo, we studied a nude mice xenograft model of colon cancer. Treatment of xenografted animals with either NDAT or gefitinib significantly inhibited tumor growth. NDAT at 1 mg/kg yielded an inhibition rate of 83%, comparable to gefitinib at 60% at 10 mg/kg. The combination treatment induced slightly more antitumoral effect with growth inhibition rates of 85.76% for NDAT (1 mg/kg) + gefitinib (10 mg/kg).

In summary, NDAT inhibited the constitutive or inducible activation of PI3K in colorectal cancer cells, which plays an important role to knock down the sialylation of EGFR to inhibit cancer cell proliferation. In addition, it enhanced gefitinib-induced antiproliferation in both gefitinib-sensitive and gefitinib-resistant colorectal cancer cell lines.

## Discussion

EGFRs may be targeted with specific antibodies or with EGFR-directed TKIs. Patients who are particularly well-suited for therapy with EGFR TKIs are those with EGFR phosphorylation-dependent cancer cell proliferation and metastasis. Therefore, inhibition of the activation of EGFR by TKIs has represented an anticancer advance. Combined treatment with EGFR-specific inhibitory agents has been shown by others to augment the antitumor response over that realized with a single EGFR inhibitor [31]. Treatment of cancers with multiple agents has been practiced for years, sometimes achieving efficacy that exceeds that of single agents. The enhancement by atorvastatin (5 μM) of gefitinib cytotoxicity through concomitant inhibition of Akt and ERK activity is an illustration [12]. Of course, toxicity may be additive in combination therapy.

The effect of gefitinib is less effective in colorectal cancer than in other types of cancer [28]. In contrast to its use in NSCLC, gefitinib administered to patients with colorectal cancer in phase I trials achieved stable disease, but no objective response in tumor size. This was the case even when patients received higher doses of the drug than doses used in lung cancer [28]. In the current preclinical studies, NDAT enhanced gefitinib-induced antiproliferation in colorectal cancer cells. The combination treatment of NDAT and gefitinib inhibited the expression of genes that are biomarkers of proliferation.

Although inhibition of ST6Gal1 expression has been reported to increase tumor cell proliferation and tumor growth in vitro and in vivo [10], our studies showed that NDAT decreased ST6Gal1 expression and cancer cell proliferation. Others have shown that decreased ST6Gal1 may increase EGF-induced EGFR phosphorylation and ERK1/2 activation in colon cancer cells [10]. Moreover, ST6Gal1 induced sialylation of both wt and mutant EGFR. ST6Gal1-induced α2,6 sialylation has been shown to be critical for adhesion and migration of colon cancer cells [10]. It is important to point out that the actions of NDAT on gene expression are highly specific, that is, they involve specific up- or downregulation of specific genes in integrin αvβ3-expressing cancer cells [24, 25, 29, 32], resulting in disruption of the cell cycle, in apoptosis and in antiangiogenesis [32]. It is thus not surprising to find specific actions of NDAT on PI3K and SG6Gal1 in colorectal cancer cells, as reported here. Nonmalignant cells express little αvβ3 and their proliferation is unaffected by NDAT [23].

In our studies, ST6Gal1 induced sialylation of mutant EGFRs (HCT116 cells). Importantly, the anticancer effect of the EGFR kinase inhibitor, gefitinib, was increased in ST6Gal1-deficient colon cancer cells. In contrast, overexpression of ST6Gal1 decreased the cytotoxic effect of gefitinib [10]. ST6Gal-mediated sialylation of EGFR has been shown to cause chemoresistance to gefitinib in colon cancer cells [10]. These findings indicate that sialylation of EGFR affects EGF-mediated cell growth and induces chemoresistance to gefitinib in colon cancer cells. Our results indicate that NDAT reduced the expression of ST6Gal1 and induced antiproliferation in both HT-29 and HCT116 cells.

By interrupting the K-ras/PI3K and K-ras/Raf complexes, gefitinib suppresses the activities of Akt and ERK [12], which leads to reduction of the synthesis of MMPs [20, 33]. Results indicated that 1 μM gefitinib did not inhibit PI3K activation in HCT116 cells although the gefitinib-induced inhibitory effect on the complexing of K-ras/PI3K and K-ras/Raf has been observed in co-mutant K-ras/PTEN or K-ras/PIK3CA NSCLC cells [12]. Persistent activity of the PI3K/Akt and/or Ras/ERK pathways is associated with gefitinib resistance in NSCLC cell lines [30]. Gefitinib, in combination with lovastatin effectively downregulated ras protein and suppressed Raf, ERK1/2, Akt, and EGFR phosphorylation in gefitinib-resistant A549 and NCI-H460 human NSCLC cells [12]. In the current studies, NDAT inhibited the activation of PI3K in mutant K-ras colorectal cancer cells, which are resistant to gefitinib treatment.

Fibronectin (FN)-induced activation of ERK, p38, Akt, cell proliferation, and invasion is mediated, at least in part, by integrins, ADAM, and EGFR. However, that gefitinib inhibits FN-induced activation of ERK, p38, and Akt, as well as cell proliferation and invasion in hepatocellular carcinoma CBO140C12 cells, indicates that these FN responses are mediated by EGFR tyrosine kinase [34].

Gefitinib has been shown to inhibit cancer metastasis. Blockade of FN-induced signaling may play a role in gefitinib-induced antimetastatic activity [34]. This TKI inhibits the formation of metastatic lesions in murine hepatocellular carcinoma [34], cell migration in mesothelioma [35], and metastasis of human chondrosarcoma cells [18]. This occurs via inhibition of expression of metastasis-related proteins, MMP-9 [18, 36], MMP-2 [18], and bFGF [18]. NDAT is known to inhibit the expression of *MMP-2*, *MMP-9*, and *VEGF-A* [23, 24, 29] and has been shown in the present studies to enhance the inhibitory effect of gefitinib on expression of *MMP-2*, *MMP-9*, and *VEGF-A*. These reports, together with our results, indicate that gefitinib at lower doses may be capable of preventing tumor metastasis in HT-29 cells. NDAT inhibits angiogenesis and it is not surprising that NDAT inhibited metastasis-related gene expression in the current studies. Studies elsewhere also have shown that SW480 colorectal carcinoma cells with ST6Gal1-knockdown are sensitive to gefitinib [10]**.**

In conclusion, we show that NDAT potentiated the antiproliferative actions of gefitinib in two colorectal cancer cell lines, HCT116 and HT-29 cells. Gefitinib and NDAT both induced antiproliferation in wt K-ras HT-29 cells. In contrast, gefitinib was unable to induce antiproliferation in K-ras mutant HCT116 cells, but NDAT enabled antiproliferation induced by gefitinib via inhibition of ST6Gal1 activity and PI3K activation. By the inhibition of crosstalk between ST6Gal1 and PI3K, NDAT enhanced gefitinib-induced antiproliferation in all cancer cell lines that were studied. Therefore, the prospect exists of use of NDAT in colorectal cancer cells, either alone or in combination with gefitinib in human gefitinib-resistant colorectal tumor cells.
